# Epitranscriptomic modifications for enhancing abiotic stress resistance in plants

**DOI:** 10.3389/fpls.2025.1538664

**Published:** 2025-05-23

**Authors:** Yunmin Zeng, Abid Muhammad, Lingyun Wan, Chenlu Gao, Pinyao Zhao, Ahmed H. El-Sappah

**Affiliations:** ^1^ Faculty of Quality Management and Inspection & Quarantine, Yibin University, Yibin, Sichuan, China; ^2^ Key Laboratory of Treatment for Special Wastewater of Sichuan Province Higher Education System, College of Chemistry and Materials Science, Sichuan Normal University, Chengdu, China; ^3^ Guangxi Key Laboratory of High-Quality Formation and Utilization of Dao-di Herbs, Guangxi Botanical Garden of Medicinal Plants, Nanning, China; ^4^ School of Agriculture, Forestry and Food Engineering, Yibin University, Yibin, Sichuan, China; ^5^ Department of Genetics, Faculty of Agriculture, Zagazig University, Zagazig, Egypt

**Keywords:** RNA mutations, epigenetics, N6-methyladenosine, environmental stress, stress-responsive genes

## Abstract

Climate change significantly impedes agricultural growth, development, and production. Plants adapt to environmental changes via the plasticity given by essential genes, which are regulated at the post-/transcriptional level. Gene regulation in plants is a complex process governed by various cellular entities, including transcription factors, epigenetic regulators, and non-coding RNAs. Successful studies have confirmed the function of epigenetic alterations such as DNA methylation/histone modification) in gene expression. In recent years, a highly specialized science known as “Epitranscriptomics” has emerged. Epitranscriptomics studies post-transcriptional RNA chemical alterations seen in all living organisms that alter RNA’s structural, functional, and biological properties. Our minireview interpreted about understanding the molecular pathways: RNA changes and stress-responsive gene regulation. Additionally, the interplay between epitranscriptomics and other regulatory levels has been addressed. In addition, we reviewed technical breakthroughs in epitranscriptomic research, including tools and techniques.

## 1 Introduction

Evolutionary and ecological constraints impact genome expression via complex regulatory networks. Some primary players governing gene expression dynamics include epigenetic alterations and chromatin re-modeling. The bulk of the previous five decades have seen the sluggish identification of RNA modifications owing to a lack of accurate detection technologies (Dhingra et al., 2023a). However, their finding gained momentum as molecular biology methods, next-generation sequencing technologies, and bioinformatic tools improved, making it feasible to correctly detect transcriptome-wide RNA modifications (Bharti et al., 2024). RNA modifications, including the N6-methyladenosine (m6A), offer an additional method for gene regulation, allowing plants to respond more efficiently to environmental changes by precisely modulating gene expression under stressful circumstances. These advancements sparked some fascinating research in RNA biology, culminating in the coining of the phrases “RNA epigenome” and “epitranscriptome”. RNA modification databases have catalogued about 170 chemical modifications (Goh and Kuang, 2024). There is emerging evidence that the epitranscriptome adds a layer to the gene regulation network for plant growth and stress responses (Xie et al., 2023).

Plant hormones are signaling chemicals that play important roles in growth, development, and environmental stress responses. Plant hormones such as abscisic acid (ABA), auxin, brassinosteroid, cytokinin, ethylene, gibberellin, jasmonate, salicylic acid, and strigolactone are well-known plant growth regulators that mediate environmental adaptations (Waadt et al., 2022). RNA modifications and plant hormone signals together regulate stress responses by modulating gene expression, ensuring the appropriate genes are activated during adverse conditions. Abiotic stressors such as drought, salt, heat, and floods are becoming more difficult for plants as the climate changes. Abiotic plant stressors reduce energy supply by limiting photosynthesis and energy-releasing catabolic processes (Li et al., 2020). Climate change and abiotic factors may exacerbate plant diseases (Chaudhry and Sidhu, 2022). Such frightening circumstances need novel methods. Recent developments in plant biology have provided critical new insights into how plants detect and react to abiotic stressors. Core stress-signaling pathways include protein kinases similar to yeast sucrose non-fermenting 1 (SNF1) and mammalian AMP-activated protein kinase (AMPK), indicating stress signaling in plants developed from energy sensing (Zhu, 2016). Stress signaling controls proteins involved in ion and water transport, metabolism, and gene expression reprogramming to achieve ionic and water balance and cellular stability under stressful circumstances (Raza et al., 2023). Stress sensing is typically likened to ligand perception hence it is commonly assumed to take place on the cell surface or at the membrane. The signal would then be sent to multiple subcellular sites, including the nucleus (Ding et al., 2024). Under salt stress, the receptor-like kinase FERONIA may detect salinity-induced cell wall defects and generate cell-specific [Ca^2+^]cyt signals to preserve cell wall integrity (Baez et al., 2022). A hydraulic signal leads to a quick root-to-shoot water deprivation signal, which triggers ABA production in Arabidopsis leaves and stomatal closure (Christmann et al., 2007). Changes in gene expression mediate phytohormones’ effects. Recently, mRNA decay has emerged as another mechanism that contributes to abiotic stress responses (Zhang et al., 2023). mRNA decapping is the process by which mRNA molecules degrade. This process involves modifications in RNA, namely m6A. These modifications can designate certain RNA molecules for degradation, hence regulating their stability and the expression of stress-related genes. Recent research indicated that the NAD^+^-capped transcriptome undergoes considerable alterations in response to ABA and that many ABA-induced transcripts lose their NAD^+^ caps after ABA treatment, perhaps boosting their stability (Borbolis and Syntichaki, 2022).

Transcriptional induction and repression are slower and endure longer than post-translational reactions involving pre-existing proteins, which occur within seconds (Millar et al., 2023). The post-transcriptional regulation level may be best for transitory stress adaption and recovery, which generally take minutes. Quantitative metrics of RNA stability are among the hardest to develop in post-transcriptional regulation. A regulatable promoter may regulate single genes and quantify degradation following transcriptional shut-off (Hernández-Elvira and Sunnerhagen, 2022). Plants use post-transcriptional regulation events as an understudied upfront regulatory switch to defend against environmental stress. Multiple constitutively present proteins are activated or deactivated by posttranslational modifications (PTMs) induced by one or more stimuli. Oxidative stress affects protein phosphorylation, sumoylation, ubiquitinylation, cysteine oxidation, glutathionylation, and methionine oxidation (García-Giménez et al., 2021).

We hypothesize that RNA modifications, particularly m6A and NAD^+^-capping, act as dynamic regulatory switches that integrate environmental signals with post-transcriptional gene regulation, enabling rapid and precise stress responses in plants. Our mini-review focused on epitranscriptomics and stress resistance: a paradigm shift. We also discussed understanding the molecular pathways: RNA alterations and stress-responsive gene regulation. Furthermore, the relationship between epitranscriptomics and various regulatory levels has been explored. Moreover, we examined technical developments in epitranscriptomic research, including tools and approaches.

## 2 Epitranscriptomics and stress resistance: a paradigm shift

### 2.1 Types of RNA modifications and their roles in stress responses

**Figure 1:**
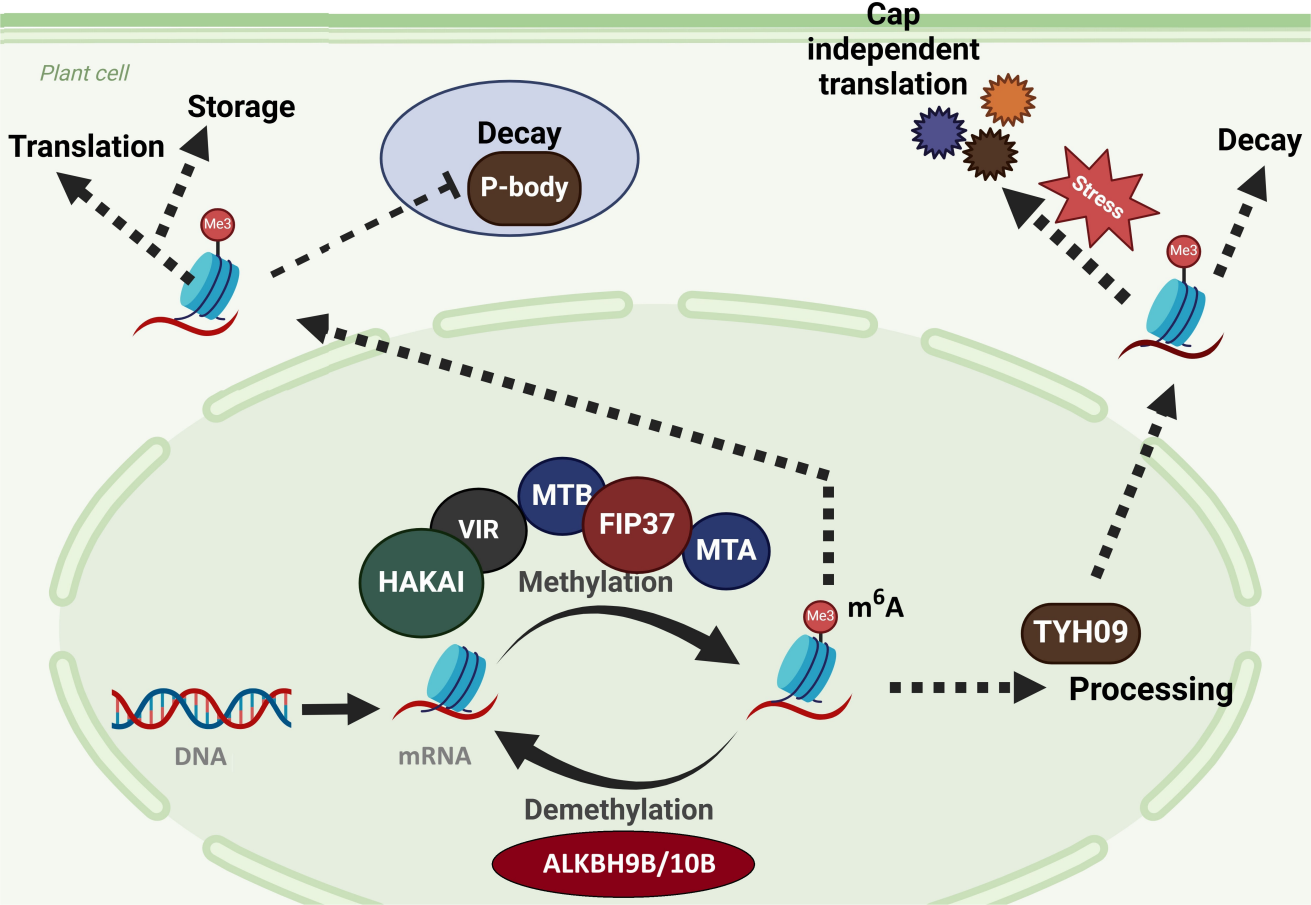
M6A RNA methylation affects a variety of biological activities. M6A RNA methylation influences mRNA splicing in the nucleus as well as varied RNA metabolism in the cytoplasm, such as cap-dependent and cap-independent translation, RNA decay in the cytosol and P-body, and RNA storage. These cellular activities rely heavily on certain “reader” proteins that recognize m6A tags on mRNAs. Writers (MTA, MTB, FIP37, VIR, and HAKAI), erasers (ALKBH9B/10B), and readers (YTH09) found in Arabidopsis (Hu et al., 2019).

RNA mutations regulate plant responses to heat, drought, salinity, and cold. The m6A, 5-methylcytosine (m5C), 7-methylguanosine (m7G), and pseudouridine (P) are important for gene expression and plant life (Ramakrishnan et al., 2022). The change must be enforced and controlled. The m6 mutation is one of the most studied plant RNA mutations. It activates *METTL3*, *METTL14*, and other methyltransferases by cleaving RNA adenine residues (Zhou et al., 2022b). The m6A alterations have a crucial role in regulating several epigenetic processes. They influence chromatin remodeling, histone modification, and DNA methylation (Zhao et al., 2021). For instance, m6A methylation facilitates the recruitment of RNA-binding proteins, including YTH-domain proteins, which regulate the processing, stability, and degradation of stress-related RNA (**Figure 1**). Moreover, m6A modifications can affect the accessibility of transcription factors to RNA by modifying its secondary structure, referred to as the “m6A switch.” This facilitates the regulation of gene expression in response to stress (Chokkalla et al., 2020). The M6A mutation affects the stability, translation, and splicing of RNA. In response to heat and drought, M6A controls the expression of stress-responsive genes (Sun et al., 2024). During heat stress in plants, m6A methylation speeds up mRNA degradation and dictates the stress response pathways to cope with high temperatures (Wang et al., 2021). These modifications also influence the RNAs associated with chromatin, facilitating the organization of chromatin structure and ensuring the regulation of critical stress-related genes following environmental conditions. Furthermore, *METTL3* and *METTL14* collaborate with proteins that alter histones and reorganize chromatin, establishing feedback loops that meticulously regulate gene expression during extended stress durations (Wu et al., 2024a). This adaptable regulation enables plants to maintain equilibrium and stability even under adverse conditions.

In addition, M6A also affects nuclear transfer RNA, ensuring that transcripts linked to stress are converted into proteins (Sun et al., 2023b). In m5C mutations, methylation of cytosine residues affects translation and RNA stability. In response to stress, m5C methylation is regulated by NSUN2 methyltransferase, regulating stress-induced gene expression in Arabidopsis (Lu et al., 2024). At high temperatures, heat stress increases m5C levels in rice, which regulates chloroplast activity and ROS production to control oxidative stress (Tang et al., 2020). This implies that the m5C mutation is crucial in protecting plants from heat damage and regulating cellular processes. In the mRNA’s 5’ cap, the m7G mutation depicts the RNA stability and translation initiation (Boo and Kim, 2020). The mRNA stability and ribosomal binding at the m7G level is important for translating stress-related proteins.

Plants adjust m7G ​​levels to adapt to unfavorable conditions during heat and drought efficiently translating stress genes (Yang et al., 2022). Under stress conditions, especially when failing due to the shortening of resources and energy, these changes are critical for maintaining metabolism and protein synthesis. Finally, to stabilize and translate RNA, P modification reverses the uridine residue (Monroe et al., 2024). Dhingra et al. (2023b) stated that pseudouridine mutation impact stress tolerance and stress-related gene transcription in plants impact stress tolerance and stress-related gene transcription in plants impact stress tolerance and stress-related gene transcription in plants impact stress tolerance and stress-related gene transcription in plants. Research indicates that stress enhanced the pseudouridine levels, which increased the production of defense proteins (Zu et al., 2023).

### 2.2 Comparative analysis of epitranscriptomic modifications under different abiotic stresses

Epitranscriptomics is critical for understanding chemical changes in RNA molecules and how plants react to environmental stresses such as drought, heat, cold, and salt. Transcripted RNAs such as m6A, m5C, m7G, and Ψ help control plant gene expression and encourage proper stress responses. A study evaluating these alterations under various stressors demonstrates both typical and common stress response patterns.

(Tan et al., 2023; Wang et al., 2023). Numerous studies have examined how m6A, one of the plants’ most significant RNA modifiers, responds to biological stress. As a key regulator of gene expression, it regulates RNA integrity, splicing, translation, and degradation (Zhou et al., 2022a; Hu et al., 2024). Chen et al. (2023) found that M6A suppresses stress-related mRNAs, which prevents protein synthesis and encourages heat-induced protein breakdown. M6A mutations suppress drought genes by changing RNA induction and expression, enabling plants to withstand water stress (Zheng et al., 2023).

In cold temperatures, M6A methylation helps the body cope with unfavorable conditions by increasing the resistance of cells. The cleaves of cytosine residues by m5C is another important mechanism for RNA synthesis and modification. In Arabidopsis and rice mutants, m5C contents are affected by heat and drought stress and are genetically influenced (Li et al., 2023a). Rice plants worldwide increase m5C during heat stress, hence regulating ROS activity and chloroplast, which are important for heat stress (Tang et al., 2020). However, m5C methylation and mRNA changes decreased, while in response to stress, m5C activity increased (Shi et al., 2022). The 5’m7G mutation is responsible for termination, translational repression, and mRNA processing (Olufunmilayo and Holsinger, 2023). Stress-associated protein m7G is induced to proliferate in response to environmental stress. The level of m7G increased due to drought and high temperatures, producing toxic proteins (Liu et al., 2024).

Besides, pseudouridine (Ψ) alters RNA structure, gene integrity, and transcription may be degraded. The increase in Ψ produces protective proteins, indicating that they work under stress (Mehrotra et al., 2023). These RNA types control gene expression and preserve cellular homeostasis according to analogies with biological systems. At high temperatures and drought, m5C and m6A are closely related to gene integrity and transcription, while Ψ and m7G mutations are important for synthesizing protein under stress (Dhingra et al., 2023a). These findings indicate that RNA modification is a regulatory mechanism in plants that enables them to adapt to environmental changes.

## 3 Deciphering the mechanistic pathways: RNA modifications and stress-responsive gene regulation

### 3.1 How RNA methylation impacts key stress-responsive genes

Stress genes are regulated by RNA methylation; m6A and m5C are particularly affected during stress.

These changes influence the RNA stability, transcription, splicing, and degradation influence RNA stability transcription, splicing, and degradation influence RNA stability, transcription, splicing, and degradation, helping plants cope with different environmental stresses. Plant RNA changes, such as m6A methylation, are frequent and well-known. A methyltransferase complex triggers it, including METTL3, METTL14, and some other proteins. In m6A mutant, gene expression is greatly influenced by RNA stability and translation efficiency. In heat stress, M6A methylation causes the loss of neutral mRNAs, thus decreasing protein synthesis and enhancing the stress gene transcription involving heat shock proteins (Zhao et al., 2020). n *Arabidopsis* stress-related mRNAs and heat shock factors (HSFs) are activated by M6A mutations, thus improving soil health (Shakespear et al., 2024). M6A methylation is crucial for assisting organisms in managing stress. It regulates the synthesis and stability of heat shock proteins (HSPs) (Sun et al., 2024). These proteins safeguard cells during periods of stress. During heat stress, M6A methylation reduces the levels of less critical mRNAs, enabling the cell to prioritize the synthesis of stress-related genes such as HSPs to mitigate damage (Wang et al., 2021). Similarly, under conditions of water scarcity, M6A methylation enhances the expression of drought-responsive genes, hence aiding plant survival in arid environments. Plants respond to water constraints through induced M6A methylation by allowing seeds to resist drought stress during germination. M5C methylation functionalizes m6A translation under stress, and the stability of RNA is stabilized (Sun et al., 2023a). The stress greatly mobilizes the methylation of m5C cytosine residues. Rice chloroplast activity and ROS generated under heat stress are functionalized by M5C methylation, thus enhancing heat tolerance (Tang et al., 2020). M5C methylation is reduced by drought stress in Arabidopsis; consequently, the stability of mRNA is affected in response to stress, indicating the impact of m5C methylation on stress-responsive genes in plants and their adaptation to the environment (Miryeganeh, 2021). M5C methylation enhances m6A by stabilizing stress-related messages and facilitating their translation into proteins (Dhingra et al., 2023a). This collaboration is crucial when plants encounter adverse conditions such as drought or saline soil. Through meticulous regulation of stress-related genes, plants may acclimate and maintain functionality. Interestingly, the RNA structure, stability, and translation are improved by changes in m6A and m5C and RNA methylation. These genes are capable of controlling the stress response in plants. In response to environmental changes, plants modify their genes respectively. In the battle against mutations and chronic stress, methylation regulates RNA, enabling plants to cope with drastic changes.

### 3.2 Interaction between epitranscriptomic marks and transcription factors in stress adaptation

Stress-induced alterations in plants are regulated by transcripts and signals from epitranscriptomes. Together, epitranscriptomics, which controls RNA molecules, and transcriptomics, which modulates gene expression, create a network that allows plants to resist abiotic conditions such as heat, cold, salt, drought, etc. By turning stress-responsive genes on or off at the appropriate moment, this link enables plants to survive in hostile surroundings. mRNA stability, synthesis, translation, and decrease, therefore impact cell stability via means of epitranscriptomic signals, including m6A, m5C, and m7G.

Among the many stress responses, including cold, drought, and heat, the plant RNA regulator m6A regulates M6A methylation, which modulates mRNA stability and transcription, impacting stress-related genes. By increasing the degradation of redundant mRNAs and creating HSPs, M6A methylation lowers cell damage during heat stress (Laosuntisuk and Doherty, 2022). In this system, HSF primarily activates *HSP* genes. M6A methylation controls HSF transcript availability as well as plant responses to extreme temperatures. M6A methylation regulates the activation of HSF genes in plants such as rice and maize (Zhao et al., 2020). This enables plants to endure extreme heat by activating pathways that react to elevated temperatures. M5C likewise controls gene methylation in response to drought stress and salt. M5C methylation controls Arabidopsis’s induced drought stress-induced stress response transcription factor DREB2A’s mRNA stability and expression. This effect has also been found in specially modified wheat designed to survive drought conditions. m5C controls the *DREB2A* gene, hence sustaining the activation of stress-response genes (Shakespear et al., 2024). This enables the wheat to endure more effectively during periods of little water. Active during a drought, these transcripts are regulated by the *m5C* gene (Chung et al., 2022).

Plant stress results from TFs turning genes on or off. It has been shown that RNA-binding proteins alter RNA interactions, influencing TF-epitope interactions. YTHDF (a YTH family protein) and other RNA-binding proteins identify M6A-modified RNA, which then interacts with transcription factors to influence genes (Liao et al., 2022). This interaction speeds up the transcriptional response to environmental stimuli, raising stress gene expression. It is important for gene activation, and the m7G mutation in mRNA’s 5’ cap causes a disturbance of translation start. Under stress, pseudouridine (Ψ) increases RNA stability and expression and modulates gene expression (Jalan et al., 2024). Epitranscriptomic and transcriptomic methods should, eventually handle gene control in stress adaption. Epitranscriptomic alteration changes RNA stability, splicing, translation, and RNA degradation to enable quick and effective responses to environmental stimuli by modifying transcription factors. This complicated relationship allows plants to change their DNA and survive in several surroundings.

## 4 Cross-talk between epitranscriptomics and other regulatory layers

### 4.1 Integration with miRNA and non-coding RNA networks in stress responses

The stress responses are partly controlled by networks of non-coding RNAs (ncRNAs) and epitranscriptomic miRNA-induced changes. There are several RNA modifications, such as m6A and m5C, which modify the activity of long non-coding ribonucleic acid (lncRNA), microRNAs (miRNA), and other ncRNAs involved in gene regulation (Sun et al., 2023a). This makes different abiotic stresses such as temperature, salinity, and drought possible to face. Microrna is a small, short RNA molecule that modulates gene expression by diminishing the level of the cognate messenger RNA through translation repression or degradation of the mRNA. mRNA editing that is dependent on m6A likewise affects mRNA and miRNA. For example, the genetically encoded m6a polymorphism has been associated with altered stability and regulation of miRNA-targeted genes that control drought-tolerant phenotypes (Hardy and Balcerowicz, 2024). M6A methylation on miRNA precursors significantly influences the activity of stress-related transcription factors, altering splicing, biosynthesis, and the maturation of miRNAs (Cai et al., 2024). This underscores the critical role of m6A in regulating stress-related signaling pathways. *M6A* methylation on precursors of miRNA dramatically influences the expression of stress-regulated transcription factors that can modify splicing, biosynthesis, and mature miRNA production (Hu et al., 2024). Other functions of non-coding RNAs also include efforts to regulate excessive stress responses like lncRNAs, which seem to be under stress themselves (Miguel et al., 2020). These mRNAs, such as *m6A* and *m5C* affect the lncRNA production due to altered dynamics of that lncRNA, affecting many other transcription factors and RNA binding proteins. Also, different types of mutations like M6A alter lncRNA dynamics and binding with transcription factors and create stress on other RNA types (Akhtar et al., 2021).

On the other hand, thermal stress for m6A mutant lncRNA encourages the expression of abnormal proteins among other stress-related genes through mRNA and miRNA (Huang et al., 2020). The relationship between RNA alterations, such as m6A, and how stress-related genes are regulated by miRNA and non-coding RNAs reveals how crucial RNA-based regulation is in stress signaling (Andersen, 2024). The armies consist of RNA modifiers, that is, miRNA and non-coding RNA, which make it possible for the attacked plants to combat environmental stresses critical for plants’ growth and development phases.

### 4.2 Potential interactions with epigenetic modifications and signaling pathways

The interaction between the epitranscriptome and the epigenetic modifications is vital to the regulation layer of plant stress responses. These two regulatory strategies act on the integrated stress response via acting on the transcription level acting on the transcription level, RNA post-transcriptional modification, and translation. They are also related to several signaling pathways that determine how plants perceive and respond to various environmental adversities, e.g., heat, drought, and salt. Methylation of adenosine at position N6 and methylation of cytosine at position C5, which are examples of epitranscriptomic modifications, affect the structure, stability, and translation of RNA molecules, while modifications such as DNA methylation, histone modifications, and chromatin remodeling are epigenetic modifications (Patrasso et al., 2023). The interaction of these two levels of regulation makes it possible to modulate the gene expression, leading to a coordinated response to stress. For instance, DNA methylation within the promoter regions of genes can inhibit gene expression when plant leaves are experiencing drought stress; intuitively, m6A changes in such genes’ mRNA molecules would alter the transcriptional stability, biasing the gene to a rapid response instead of waiting for normal stress response times (Liu et al., 2023).

Alterations in chromatin structure, such as histone acetylation and methylation, can drive genes into states where they become more responsive to stress at a given time or are no longer fit for the transcriptional machinery. For instance, heat stress activates the nucleus, which later leads to the appearance of various stress-shock proteins as well as HSPs – proteins involved in heat stress management. It was demonstrated that m6A mutations impact HSF transcript stability as well as translation rates, initiating a quicker stress response (Lamelas et al., 2022). Fan et al. (2024) demonstrated that the simultaneous blockage by targeting many genes encoding for ABA proteins as well as m6A-assisted epitranscriptomic alterations conceivably creates a close connection between ABA production and drought and salinity stresses. Further, stress-induced genes and proteins, through miRNAs that are modulated by RNA modifications together with epitranscriptome, link all these pathways, bridging the epitranscriptomic and epigenetic levels within signaling networks.

## 5 Technological advances in epitranscriptomic research: tools and approaches

Collaboration between epitranscriptomic alterations and epigenetics is critical in plant stress control.

Two levels of regulation mediate stress-induced responses by influencing gene expression at three levels: transcriptional, post-translational, and translation, also involving various signaling pathways. That controls how plants sense and respond to environmental stresses such as heat, drought, and salinity. Epitranscriptomic modifications such as m6A and m5C affect RNA function, stability, and translation, while epigenetic modifications such as DNA methylation histone modifications and deciphering the regulation of chromatin remodeling. The interaction of these two regulatory levels positively affects gene expression, causing it to increase. For example, in drought-prone species, DNA methylation of promoter regions reduces gene expression. However, m6A changes in the mRNA of the same gene enhance or regulate translation. This leads to a rapid stress response (Malik et al., 2024). At the molecular level, these alterations facilitate rapid adaptation to stress in organisms by meticulously regulating gene expression through many interconnected mechanisms (Turner, 2009). DNA methylation silences genes that might impede stress management, whereas m6A methylation modulates RNA stability and the efficiency of translating stress-related genes into proteins (Yang and Chen, 2021). During a drought, DNA methylation can inhibit the activity of non-essential genes, whereas m6A methylation enhances the expression of proteins from critical stress-related genes (Li et al., 2023b). This enables the organism to utilize its resources efficiently and adapt rapidly. Collectively, these mechanisms ensure that the stress response is accurate and energy-efficient in confronting challenging environmental situations.

Histone modifications such as acetylation and methylation can change the structure of chromatin. This makes the gene less sensitive to the transcription mechanism, while m6A can increase or decrease the stability of the mRNA produced by the gene. The radioactivity that controls different stress responses is linked with these changes. For instance, the HSF signaling pathway is activated by heat stress, which provokes the transcription of the HSPs responsible for protein folding. Previous studies have found that the stability and localization of HSF are greatly affected by m6A mutations, causing a quick and efficient response (Huang et al., 2020). Similarly, ABA signaling, crucial for drought and salinity, responds to modification in epitranscriptomics like m6A to control expression in response to ABA (Hu et al., 2024).

## 6 Future directions: harnessing epitranscriptomics for crop improvement

Epitranscriptomics has great enormous potential for plant evolution. This is particularly important in reproduction and in regulating environmental stresses such as heat, cold, drought, salt and nutritional deficiency. The role of RNA transcripts is an important future direction in stress-induced genes. The identification of specific RNA mutations such as m6A, m5C, and m7G and their role in stress regulation raises the possibility of using these markers to increase plant stress tolerance, such as the m6A mutation, which has been shown to affect the translation and stability of stress-related genes. Additionally, to manipulate RNA editing enzymes, gene editing tools such as CRISPR/Cas9 offer excellent opportunities to achieve desired results. The scientists created high-stress tolerance plants by inhibiting methylate or demethylate RNA enzymes, such as TET for m5C methylation and METTL3 for m6A methylation. This strategy can enhance plant growth under abiotic stress without requiring an incubation period (Wu et al., 2024b). Data aggregation could be another interesting method.

Epitranscriptome fits well with other omics techniques such as proteomics, metabolomics, bioinformatics, etc. These techniques could be applied along with epitranscriptome to better understand how plants respond to different stresses. Hence, it provides deep insights into the molecular mechanisms of stress tolerance. The latest bioinformatics tools help researchers in new RNA mutations discoveries and their possible role in stress responses. The important breeding targets have been acquired by developing new RNA-based mutation techniques.

Furthermore, a CRISPR-Cas13-based targeted RNA methylation/demethylation system capable of effectively altering a single mRNA base would considerably expedite research into the relationships between m6A modification and epigenetic regulators (Hu et al., 2024). This technique employs a guide RNA to target the Cas13 protein to certain RNA sequences. Conjugating Cas13 with enzymes that methylate or demethylate may accurately modify m6A sites by adding or removing methyl groups at designated locations (Tang et al., 2021). CRISPR-Cas13 employs specific techniques to enhance precision to prevent unintended alterations during RNA editing (Kordyś et al., 2022). It uses computational approaches to generate highly specialized guide RNAs, minimizing errors. By examining the conformation and sequence of the guide RNA and its alignment with the target RNA, scientists can ensure it exclusively binds to the correct locus. Furthermore, employing enhanced variants of Cas13 that exhibit greater precision minimizes mistakes, ensuring modifications occur exclusively at the designated m6A loci (Wilson et al., 2020). This precision enables researchers to examine the influence of m6A changes on gene expression, RNA stability, and protein synthesis. Nonetheless, there is potential for inadvertent alterations since the guide RNA may occasionally attach to incorrect RNA sequences, resulting in undesired mutations (Aledhari and Rahouti, 2024). To address this issue, computer systems employing machine learning and artificial intelligence are being utilized in the design process to anticipate and eliminate off-target areas. These methods evaluate several parameters, including sequence similarity and stability, to enhance the design of guide RNAs for improved accuracy. Advanced computational methods are employed to enhance the design and precision of the guide RNA, therefore mitigating these concerns (Zhang et al., 2022). The combination of bioinformatics and artificial intelligence techniques has the potential to change plant abiotic stress management.

Artificial intelligence (AI)-powered technologies have been employed to analyze extensive datasets of gene activity to identify genes and networks that react to stress (Mentis et al., 2024). Techniques such as Genome-Wide Association Studies (GWAS) utilizing bioinformatics have facilitated the identification of genetic markers associated with the drought and salinity tolerance of crops like rice and wheat (Huang, 2024). Scientists can acquire new insights into stress reactions by merging multi-omics data and employing powerful AI algorithms. An example is employing machine learning to forecast plant responses to adverse conditions, thus facilitating the development of stress-resistant crops. Instruments such as drones and sophisticated imaging systems, augmented by AI, provide real-time monitoring of plant responses to heat and drought (Walsh et al., 2024). Finally, modern scientific approaches such as CRISPR-Cas systems, RNA interference, high-throughput sequencing technologies, and transcriptome editing platforms in plant breeding and genetics brought significant opportunities for agricultural production and resilience to climate change via RNA manipulation and the integration of outcomes by cutting-edge biotechnological methods. CRISPR-Cas systems, coupled with RNA guide designs developed through bioinformatics, have enhanced plant resilience against environmental challenges like drought and severe temperatures. Furthermore, AI-driven models analyzing RNA networks have enhanced gene-editing techniques, enabling plants to withstand stress better. Scientists have successfully created plants that thrive in harsh conditions, ensuring their safety and survival for future generations.

## References

[B1] Akhtar J. Lugoboni M. Junion G. (2021). m6A RNA modification in transcription regulation. Transcription 12, 266–276. doi: 10.1080/21541264.2022.2057177 35380917 PMC9208771

[B2] Aledhari M. Rahouti M. (2024). Gene and RNA editing: methods, enabling technologies, applications, and future directions. arXiv. preprint. arXiv:2409.09057. doi: 10.48550/arXiv.2409.09057

[B3] Andersen R. E. (2024). Long non-coding RNAs: recent insights, remaining challenges, and exciting new directions. Hum. Genet. 143, 797–799. doi: 10.1007/s00439-024-02689-8 39048854

[B4] Baez L. A. Tichá T. Hamann T. (2022). Cell wall integrity regulation across plant species. Plant Mol. Biol. 109, 483–504. doi: 10.1007/s11103-022-01284-7 35674976 PMC9213367

[B5] Bharti M. K. Chandra D. Siddique R. Ranjan K. Kumar P. (2024). “Recent advancement in high-throughput “omics” technologies,” in Current Omics Advancement in Plant Abiotic Stress Biology (Academic Press), 343–355. doi: 10.1016/B978-0-443-21625-1.00023-3

[B6] Boo S. H. Kim Y. K. (2020). The emerging role of RNA modifications in the regulation of mRNA stability. Exp. Mol. Med. 52, 400–408. doi: 10.1038/s12276-020-0407-z 32210357 PMC7156397

[B7] Borbolis F. Syntichaki P. (2022). Biological implications of decapping: beyond bulk mRNA decay. FEBS J. 289, 1457–1475. doi: 10.1111/febs.v289.6 33660392

[B8] Cai J. Shen L. Kang H. Xu T. (2024). RNA modifications in plant adaptation to abiotic stresses. Plant. Commun. 6 (2), 101229. doi: 10.1016/j.xplc.2024.101229 39709520 PMC11897461

[B9] Chaudhry S. Sidhu G. P. S. (2022). Climate change regulated abiotic stress mechanisms in plants: A comprehensive review. Plant. Cell. Rep. 41, 1–31. doi: 10.1007/s00299-021-02759-5 34351488

[B10] Chen B. Yuan C. Guo T. Liu J. Yang B. Lu Z. (2023). Molecular mechanism of m6A methylation modification genes METTL3 and FTO in regulating heat stress in sheep. Int. J. Mol. Sci. 24, 11926. doi: 10.3390/ijms241511926 37569302 PMC10419070

[B11] Chokkalla A. K. Mehta S. L. Vemuganti R. (2020). Epitranscriptomic regulation by m6A RNA methylation in brain development and diseases. J. Cereb. Blood Flow Metab. 40, 2331–2349. doi: 10.1177/0271678X20960033 32967524 PMC7820693

[B12] Christmann A. Weiler E. W. Steudle E. Grill E. (2007). A hydraulic signal in root-to-shoot signaling of water shortage. Plant J. 52, 167–174. doi: 10.1111/j.1365-313X.2007.03234.x 17711416

[B13] Chung S. Kwon C. Lee J.-H. (2022). Epigenetic control of abiotic stress signaling in plants. Genes Genomics, 1–12. doi: 10.1007/s13258-021-01163-3 34515950

[B14] Dhingra Y. Gupta S. Gupta V. Agarwal M. Katiyar-Agarwal S. (2023a). The emerging role of epitranscriptome in shaping stress responses in plants. Plant Cell Rep. 42, 1531–1555. doi: 10.1007/s00299-023-03046-1 37481775

[B15] Dhingra Y. Lahiri M. Bhandari N. Kaur I. Gupta S. Agarwal M.. (2023b). Genome-wide identification, characterization, and expression analysis unveil the roles of pseudouridine synthase (PUS) family proteins in rice development and stress response. Physiol. Mol. Biol. Plants 29, 1981–2004. doi: 10.1007/s12298-023-01396-4 38222285 PMC10784261

[B16] Ding S. Chen Y. Huang C. Song L. Liang Z. Wei B. (2024). Perception and response of skeleton to mechanical stress. Phys. Life Rev. doi: 10.1016/j.plrev.2024.03.011 38564907

[B17] Fan S. Zhang Y. Zhu S. Shen L. (2024). Plant RNA-binding proteins: phase-separation dynamics and functional mechanisms underlying plant development and stress responses. Mol. Plant. doi: 10.1016/j.molp.2024.02.016 38419328

[B18] García-Giménez J.-L. Garcés C. Romá-Mateo C. Pallardó F. V. (2021). Oxidative stress-mediated alterations in histone post-translational modifications. Free Radical Biol. Med. 170, 6–18. doi: 10.1016/j.freeradbiomed.2021.02.027 33689846

[B19] Goh W. S. Kuang Y. (2024). Heterogeneity of chemical modifications on RNA. Biophys. Rev. 16, 79–87. doi: 10.1007/s12551-023-01128-8 38495447 PMC10937866

[B20] Hardy E. C. Balcerowicz M. (2024). Untranslated yet indispensable—UTRs act as key regulators in the environmental control of gene expression. J. Exp. Bot., erae073. doi: 10.1093/jxb/erae073 PMC11263492 38394144

[B21] Hernández-Elvira M. Sunnerhagen P. (2022). Post-transcriptional regulation during stress. FEMS Yeast. Res. 22, foac025. doi: 10.1093/femsyr/foac025 35561747 PMC9246287

[B22] Hu J. Manduzio S. Kang H. (2019). Epitranscriptomic RNA methylation in plant development and abiotic stress responses. Front. Plant Sci. 10, 500. doi: 10.3389/fpls.2019.00500 31110512 PMC6499213

[B23] Hu J. Xu T. Kang H. (2024). Crosstalk between RNA m6A modification and epigenetic factors for gene regulation in plants. Plant. Commun. 10.1016/j.xplc.2024.101037 PMC11573915 38971972

[B24] Huang H. Weng H. Chen J. (2020). m6A modification in coding and non-coding RNAs: roles and therapeutic implications in cancer. Cancer Cell. 37 (3), 270–288. doi: 10.1016/j.ccell.2020.02.004 PMC7141420 32183948

[B25] Huang W. (2024). The current situation and future of using GWAS strategies to accelerate the improvement of crop stress resistance traits. Mol. Plant Breed. 15. doi: 10.5376/mpb.2024.15.0007

[B26] Jalan A. Jayasree P. Karemore P. Narayan K. P. Khandelia P. (2024). Decoding the ‘fifth’nucleotide: impact of RNA pseudouridylation on gene expression and human disease. Mol. Biotechnol. 66, 1581–1598. doi: 10.1007/s12033-023-00792-1 37341888

[B27] Kordyś M. Sen R. Warkocki Z. (2022). Applications of the versatile CRISPR-Cas13 RNA targeting system. Wiley. Interdiscip. Rev.: RNA 13, e1694. 34553495 10.1002/wrna.1694

[B28] Lamelas L. Valledor L. López-Hidalgo C. Cañal M. J. Meijón M. (2022). Nucleus and chloroplast: a necessary understanding to overcome heat stress in Pinus radiata. Plant. Cell Environ. 45, 446–458. doi: 10.1111/pce.14238 34855991

[B29] Laosuntisuk K. Doherty C. J. (2022). The intersection between circadian and heat-responsive regulatory networks controls plant responses to increasing temperatures. Biochem. Soc. Trans. 50, 1151–1165. doi: 10.1042/BST20190572 35758233 PMC9246330

[B30] Li Y. Ding L. Zhou M. Chen Z. Ding Y. Zhu C. (2023b). Transcriptional regulatory network of plant cadmium stress response. Int. J. Mol. Sci. 24, 4378. doi: 10.3390/ijms24054378 36901809 PMC10001906

[B31] Li S. Yang W. Guo J. Li X. Lin J. Zhu X. (2020). Changes in photosynthesis and respiratory metabolism of maize seedlings growing under low temperature stress may be regulated by arbuscular mycorrhizal fungi. Plant Physiol. Biochem. 154, 1–10. doi: 10.1016/j.plaphy.2020.05.025 32505784

[B32] Li J. Y. Yang C. Xu J. Lu H. P. Liu J. X. (2023a). The hot science in rice research: How rice plants cope with heat stress. Plant. Cell Environ. 46, 1087–1103. doi: 10.1111/pce.14509 36478590

[B33] Liao J. Wei Y. Liang J. Wen J. Chen X. Zhang B.. (2022). Insight into the structure, physiological function, and role in cancer of m6A readers—YTH domain-containing proteins. Cell Death Discov. 8, 137. doi: 10.1038/s41420-022-00947-0 35351856 PMC8964710

[B34] Liu J. Wu Y. Dong G. Zhu G. Zhou G. (2023). Progress of research on the physiology and molecular regulation of sorghum growth under salt stress by gibberellin. Int. J. Mol. Sci. 24, 6777. doi: 10.3390/ijms24076777 37047750 PMC10094886

[B35] Liu S. Wu H. Zhao Z. (2024). Heat stress-induced decapping of WUSCHEL mRNA enhances stem cell thermotolerance in Arabidopsis. Mol. Plant. doi: 10.1016/j.molp.2024.10.011 39468792

[B36] Lu Y. Yang L. Feng Q. Liu Y. Sun X. Liu D.. (2024). RNA 5-methylcytosine modification: regulatory molecules, biological functions, and human diseases. Genom. Proteomics Bioinf., qzae063. doi: 10.1093/gpbjnl/qzae063 PMC11634542 39340806

[B37] Malik P. Kaur K. Kaur P. Seni S. Kaur H. (2024). “Breeding climate-smart cultivars: can transcriptomics enable strategic cereal breeding?,” in Omics and System Biology Approaches for Delivering Better Cereals (CRC Press), 201–231.

[B38] Mehrotra S. Dimkpa C. O. Goyal V. (2023). Survival mechanisms of chickpea (Cicer arietinum) under saline conditions. Plant Physiol. Biochem., 108168. doi: 10.1016/j.plaphy.2023.108168 38008005

[B39] Mentis A.-F. A. Lee D. Roussos P. (2024). Applications of artificial intelligence– machine learning for detection of stress: a critical overview. Mol. Psychiatry 29, 1882–1894. doi: 10.1038/s41380-023-02047-6 37020048

[B40] Miguel V. Lamas S. Espinosa-Diez C. (2020). Role of non-coding-RNAs in response to environmental stressors and consequences on human health. Redox Biol. 37, 101580. doi: 10.1016/j.redox.2020.101580 32723695 PMC7767735

[B41] Millar S. R. Huang J. Q. Schreiber K. J. Tsai Y.-C. Won J. Zhang J.. (2023). A new phase of networking: the molecular composition and regulatory dynamics of mammalian stress granules. Chem. Rev. 123, 9036–9064. doi: 10.1021/acs.chemrev.2c00608 36662637 PMC10375481

[B42] Miryeganeh M. (2021). Plants’ epigenetic mechanisms and abiotic stress. Genes 12, 1106. doi: 10.3390/genes12081106 34440280 PMC8394019

[B43] Monroe J. Eyler D. E. Mitchell L. Deb I. Bojanowski A. Srinivas P.. (2024). N1-Methylpseudouridine and pseudouridine modifications modulate mRNA decoding during translation. Nat. Commun. 15, 8119. doi: 10.1038/s41467-024-51301-0 39284850 PMC11405884

[B44] Olufunmilayo E. O. Holsinger R. D. (2023). Roles of non-coding RNA in alzheimer’s disease pathophysiology. Int. J. Mol. Sci. 24, 12498. doi: 10.3390/ijms241512498 37569871 PMC10420049

[B45] Patrasso E. A. Raikundalia S. Arango D. (2023). Regulation of the epigenome through RNA modifications. Chromosoma 132, 231–246. doi: 10.1007/s00412-023-00794-7 37138119 PMC10524150

[B46] Ramakrishnan M. Rajan K. S. Mullasseri S. Palakkal S. Kalpana K. Sharma A.. (2022). The plant epitranscriptome: revisiting pseudouridine and 2′-O-methyl RNA modifications. Plant Biotechnol. J. 20, 1241–1256. doi: 10.1111/pbi.13829 35445501 PMC9241379

[B47] Raza A. Tabassum J. Fakhar A. Z. Sharif R. Chen H. Zhang C.. (2023). Smart reprograming of plants against salinity stress using modern biotechnological tools. Crit. Rev. Biotechnol. 43, 1035–1062. doi: 10.1080/07388551.2022.2093695 35968922

[B48] Shakespear S. Sivaji M. Kumar V. Arumugam Pillai M. Wani S. H. Penna S.. (2024). Navigating through harsh conditions: coordinated networks of plant adaptation to abiotic stress. J. Plant Growth Regul. 44, 1396–1414. doi: 10.1007/s00344-023-11224-4

[B49] Shi M. Wang C. Wang P. Zhang M. Liao W. (2022). Methylation in DNA, histone, and RNA during flowering under stress condition: A review. Plant Sci. 324, 111431. doi: 10.1016/j.plantsci.2022.111431 36028071

[B50] Sun H. Li K. Liu C. Yi C. (2023a). Regulation and functions of non-m6A mRNA modifications. Nat. Rev. Mol. Cell Biol. 24, 714–731. doi: 10.1038/s41580-023-00622-x 37369853

[B51] Sun Y. Sun Y. He X. Li S. Xu X. Feng Y.. (2024). Transcriptome-wide methylated RNA immunoprecipitation sequencing profiling reveals m6A modification involved in response to heat stress in Apostichopus japonicus. BMC Genomics 25, 1071. doi: 10.1186/s12864-024-10972-1 39528936 PMC11556200

[B52] Sun L. Zuo Z. Qiu X. Wang G. Li Q. Qiu J.. (2023b). Recent advances in the interplay between stress granules and m6A RNA modification. Curr. Opin. Solid. State. Mater. Sci. 27, 101119. doi: 10.1016/j.cossms.2023.101119

[B53] Tan Q. W. Lim P. K. Chen Z. Pasha A. Provart N. Arend M.. (2023). Cross-stress gene expression atlas of Marchantia polymorpha reveals the hierarchy and regulatory principles of abiotic stress responses. Nat. Commun. 14, 986. doi: 10.1038/s41467-023-36517-w 36813788 PMC9946954

[B54] Tang Y. Gao C.-C. Gao Y. Yang Y. Shi B. Yu J.-L.. (2020). OsNSUN2-mediated 5-methylcytosine mRNA modification enhances rice adaptation to high temperature. Dev. Cell 53, 272–286. e277. doi: 10.1016/j.devcel.2020.03.009 32275888

[B55] Tang T. Han Y. Wang Y. Huang H. Qian P. (2021). Programmable system of Cas13-mediated RNA modification and its biological and biomedical applications. Front. Cell Dev. Biol. 9, 677587. doi: 10.3389/fcell.2021.677587 34386490 PMC8353156

[B56] Turner B. M. (2009). Epigenetic responses to environmental change and their evolutionary implications. Philos. Trans. R. Soc. B.: Biol. Sci. 364, 3403–3418. doi: 10.1098/rstb.2009.0125 PMC2781845 19833651

[B57] Waadt R. Seller C. A. Hsu P.-K. Takahashi Y. Munemasa S. Schroeder J. I. (2022). Plant hormone regulation of abiotic stress responses. Nat. Rev. Mol. Cell Biol. 23, 680–694. doi: 10.1038/s41580-022-00479-6 35513717 PMC9592120

[B58] Walsh J. J. Mangina E. Negrão S. (2024). Advancements in imaging sensors and AI for plant stress detection: A systematic literature review. Plant Phenom. 6, 0153. doi: 10.34133/plantphenomics.0153 PMC10905704 38435466

[B59] Wang M.-K. Gao C.-C. Yang Y.-G. (2023). Emerging roles of RNA methylation in development. Accounts. Chem. Res. 56, 3417–3427. doi: 10.1021/acs.accounts.3c00448 37965760

[B60] Wang L. Maimaitiyiming Y. Su K. Hsu C.-H. (2021). RNA m 6 A modification: the mediator between cellular stresses and biological effects. Epitranscriptomics, 353–390. doi: 10.1007/978-3-030-71612-7_13

[B61] Wilson C. Chen P. J. Miao Z. Liu D. R. (2020). Programmable m6A modification of cellular RNAs with a Cas13-directed methyltransferase. Nat. Biotechnol. 38, 1431–1440. doi: 10.1038/s41587-020-0572-6 32601430 PMC7718427

[B62] Wu Z. Zhang W. Qu J. Liu G.-H. (2024a). Emerging epigenetic insights into aging mechanisms and interventions. Trends Pharmacol. Sci. doi: 10.1016/j.tips.2023.12.002 38216430

[B63] Wu Z. Zhou R. Li B. Cao M. Wang W. Li X. (2024b). Methylation modifications in tRNA and associated disorders: Current research and potential therapeutic targets. Cell Prolif., e13692. doi: 10.1111/cpr.13692 38943267 PMC11503269

[B64] Xie Y. Chan L. Y. Cheung M. Y. Li M. W. Lam H. M. (2023). Current technical advancements in plant epitranscriptomic studies. Plant Genome 16, e20316. doi: 10.1002/tpg2.20316 36890704 PMC12806912

[B65] Yang B. Chen Q. (2021). Cross-talk between oxidative stress and m6A RNA methylation in cancer. Oxid. Med. Cell. Longevity 2021, 6545728. doi: 10.1155/2021/6545728 PMC8416400 34484567

[B66] Yang X. Patil S. Joshi S. Jamla M. Kumar V. (2022). Exploring epitranscriptomics for crop improvement and environmental stress tolerance. Plant Physiol. Biochem. 183, 56–71. doi: 10.1016/j.plaphy.2022.04.031 35567875

[B67] Zhang J. Fei Y. Sun L. Zhang Q. C. (2022). Advances and opportunities in RNA structure experimental determination and computational modeling. Nat. Methods 19, 1193–1207. doi: 10.1038/s41592-022-01623-y 36203019

[B68] Zhang Y. Xu J. Li R. Ge Y. Li Y. Li R. (2023). Plants’ response to abiotic stress: Mechanisms and strategies. Int. J. Mol. Sci. 24, 10915. doi: 10.3390/ijms241310915 37446089 PMC10341657

[B69] Zhao Y. Chen Y. Jin M. Wang J. (2021). The crosstalk between m6A RNA methylation and other epigenetic regulators: a novel perspective in epigenetic remodeling. Theranostics 11, 4549. doi: 10.7150/thno.54967 33754077 PMC7977459

[B70] Zhao J. Lu Z. Wang L. Jin B. (2020). Plant responses to heat stress: physiology, transcription, noncoding RNAs, and epigenetics. Int. J. Mol. Sci. 22, 117. doi: 10.3390/ijms22010117 33374376 PMC7795586

[B71] Zheng H. Gao Y. Dang Y. Wu F. Wang X. Zhang F.. (2023). Characterization of the m6A gene family in sorghum and its function in growth, development and stress resistance. Industrial. Crops Prod. 198, 116625. doi: 10.1016/j.indcrop.2023.116625

[B72] Zhou L. Gao G. Tang R. Wang W. Wang Y. Tian S.. (2022a). m6A-mediated regulation of crop development and stress responses. Plant Biotechnol. J. 20, 1447–1455. doi: 10.1111/pbi.13792 35178842 PMC9342612

[B73] Zhou W. Wang X. Chang J. Cheng C. Miao C. (2022b). The molecular structure and biological functions of RNA methylation, with special emphasis on the roles of RNA methylation in autoimmune diseases. Crit. Rev. Clin. Lab. Sci. 59, 203–218. doi: 10.1080/10408363.2021.2002256 34775884

[B74] Zhu J.-K. (2016). Abiotic stress signaling and responses in plants. Cell 167, 313–324. doi: 10.1016/j.cell.2016.08.029 27716505 PMC5104190

[B75] Zu X. Luo L. Wang Z. Gong J. Yang C. Wang Y.. (2023). A mitochondrial pentatricopeptide repeat protein enhances cold tolerance by modulating mitochondrial superoxide in rice. Nat. Commun. 14, 6789. doi: 10.1038/s41467-023-42269-4 37880207 PMC10600133

